# *Camellia sinensis* L. Alleviates Pulmonary Inflammation Induced by Porcine Pancreas Elastase and Cigarette Smoke Extract

**DOI:** 10.3390/antiox11091683

**Published:** 2022-08-28

**Authors:** Dong-Uk Shin, Ji-Eun Eom, Hyeon-Ji Song, Sun Young Jung, Thi Van Nguyen, Kyung Min Lim, Ok Hee Chai, Hyun-Jin Kim, Gun-Dong Kim, Hee Soon Shin, So-Young Lee

**Affiliations:** 1 Department of Food Biotechnology, Korea University of Science and Technology (UST), Daejeon 34113, Korea; 2 Division of Food Functionality Research, Korea Food Research Institute (KFRI), Wanju 55365, Korea; 3 Food Function Infrastructure Team, Korea Food Research Institute (KFRI), Wanju 55365, Korea; 4 Department of Food Science and Technology, Jeonbuk National University, Jeonju 54896, Korea; 5 Department of Anatomy, Institute of Medical Science, Jeonbuk National University Medical School, Jeonju 54907, Korea; 6 Division of Applied Life Science (BK21 Four), Department of Food Science and Technology, Institute of Agriculture and Life Science, Gyeongsang National University, 501 Jinjudaero, Jinju 52828, Korea; 7 EZmass. Co., Ltd., 501 Jinjudaero, Jinju 55365, Korea

**Keywords:** cigarette smoke, chronic obstructive pulmonary diseases, lung inflammation, airway epithelial, alveolar macrophages, neutrophils, NF-κB, nuclear factor erythroid-2-related factor 2 heme oxygenase-1

## Abstract

Cigarette smoke (CS) is the major factor in the development of chronic obstructive pulmonary disease (COPD), the third leading cause of death worldwide. Furthermore, although *Camellia sinensis* (CN) has been known as an anti-inflammatory material, the effect of CN has not yet been known on pulmonary inflammation in COPD. Thus, we investigated the protective effects of *Camellia sinensis* L. extract (CLE) against pulmonary inflammation in porcine pancreas elastase (PPE) and a cigarette smoke extract (CSE)-induced COPD mouse model. Oral administration of CLE suppressed the symptoms such as infiltration of immune cells, cytokines/chemokines secretion, mucus hypersecretion, and injuries of the lung parenchyma. Increased inflammatory responses in COPD are mediated by various immune cells such as airway epithelial cells, neutrophils, and alveolar macrophages. Thus, we investigated the effect and mechanisms of CLE in H292, HL-60, and MH-S cells. The CLE inhibited the expression of IL-6, IL-8, MUC5AC and MUC5B on CSE/LPS-stimulated H292 cells and also suppressed the formation of neutrophil extracellular traps and secretion of neutrophil elastase by inhibiting reactive oxygen species in PMA-induced HL-60 cells. In particular, the CLE suppressed the release of cytokines and chemokines caused by activating the nuclear factor kappa-light-chain-enhancer of activated B via the activation of nuclear factor erythroid-2-related factor 2 and the heme oxygenase-1 pathway in CSE/LPS-stimulated MH-S cells. Therefore, we suggest that the CLE administration be the effective approach for treating or preventing chronic pulmonary diseases such as COPD induced by CS.

## 1. Introduction

Chronic obstructive pulmonary disease (COPD) is a common airway disease. It is the third leading cause of death worldwide, causing 3.23 million deaths in 2019 [1]. Therefore, COPD is estimated to become the main economic burden of chronic diseases in the future [1,2]. Cigarette smoking is a major predisposing factor to COPD. More than 90% of patients with COPD actively smoke or used to smoke [3]. Cigarette smoke contains numerous organic compounds such as nicotine, tar, polycyclic aromatic hydrocarbons (PAHs), volatile compounds, and heavy metals. Cigarette smoking causes cancer, heart disease, lung diseases and COPD [4]. Inflammation in COPD is mediated by various cells such as lung epithelial cells, macrophages and neutrophils [4]. Lung epithelial cells produce inflammatory mediators such as cytokines and chemokines as well as mucus and reactive oxygen species (ROS) [5]. Inflammation and mucus overproduction are complicated by the difficulty in clearing secretions because of poor ciliary function, distal airway occlusion, and an ineffective cough secondary to respiratory muscle weakness and reduced peak expiratory flow [6]. Alveolar macrophages stimulated by cigarette smoke produce pro-inflammatory cytokines, chemokines, and matrix metalloproteases which increase the infiltration of immune cells in the bronchoalveolar fluid. Neutrophils stimulated by cigarette smoke release neutrophil elastase (NE) and induce neutrophil extracellular traps (NETs). The destruction of elastin by proteases and elastases leads to the development of emphysema [6], a pathological condition associated with COPD and characterized by airway remodeling and alveolar wall destruction [7]. Cigarette smoke (CS) amplifies the inflammatory response by inducing the production of ROS, such as hydrogen peroxide and superoxide [8]. ROS activates the mitogen-activated protein kinases (MAPK) pathway in lung epithelial cells and induces mucus overproduction [9]. In addition, ROS activates the nuclear factor-κB (NF-κB) and MAPK with increasing expression levels of pro-inflammatory cytokines such as interleukin 6 (IL-6), tumor necrosis factor-α (TNF-α), and interleukin 8 (IL-8) and inhibits the phagocytosis by alveolar macrophages [10,11]. The enzymatic processing of ROS by myeloperoxidase (MPO) is vital for NET formation in neutrophils [12]. This leads to the production of pro-inflammatory cytokines and the recruitment of inflammatory cells. Therefore, long-term CS exposure worsens this disease. The suppression of the immune response mediated by CS-stimulated epithelial cells, macrophages, and neutrophils may be a target in COPD treatment.

*Camellia sinensis* (CN) is a plant species native to China, South Asia and Southeast Asia, but grown today in all tropical regions [13]. CN is used in the production of teas such as green tea, white tea, and oolong tea, but is also used as a herbal drug against obesity, cancer, and diabetes [14]. CN is reported to contain 4000 bioactive compounds, of which one third are polyphenols. Other compounds include alkaloids, amino acids, carbohydrates, proteins, volatile organic compounds, fluoride, aluminum, minerals, and trace elements. CN also contains flavonols, mainly quercetin, kaempferol, myricetin, and their glycosides [15,16]. The polyphenols found in CN were mostly flavonoids. The major lavonoids are (−)-epicatechin gallate (ECG), (−)-epicatechin (EC), (−)-epigallocatechin (EGC), and (−)-epigallocatechin gallate (EGCG) [16]. These diverse bioactive compounds show health benefits, including antioxidant, anti-inflammatory, anti-aging, anti-obesity, and anti-cancer activities [17,18,19,20,21]. Various studies on the prevention and treatment of COPD have been reported; however, studies related to the effect of CN on COPD have not been reported. As similar studies related to attenuating effects of CN on lung disease, there was a report that black tea alleviated lung injury caused by particulate matter [22]. However, black tea produced through the fermentation of CN has different composition (especially theaflavin) from CN, as well as there being numerous irritating factors in particulate matter which induced different pathological features in PPE/CSE-induced COPD. Another study related to attenuating effects of CN on lung disease has reported that tea polyphenols alleviate the lung injury of hepatopulmonary syndrome in common bile duct ligation rats through endotoxin-TNF [23]. These previous studies dealt with respiratory disease different from COPD. Therefore, in the present study, we investigated the anti-inflammatory effects and mechanisms of CN ethanol extract (CLE) in a mouse model of PPE/CSE-induced COPD and in a cell-based experimental model using H292, HL-60, and MH-S cell lines.

## 2. Materials and Methods

### 2.1. Plant Material and Preparation of Extracts

Dried *Camellia sinensis* L. was extracted by heating with 20-times volumes of 70% ethanol for 6 h. CLE was filtered through a cartridge filter using a Rotavapor R—210 (BUCHI Labortechnik AG, Flawil, Switzerland), the filtered extract was concentrated under a vacuum at 50 °C and then dried. CLE was dissolved in dimethyl sulfoxide before its use in the experiments.

### 2.2. Analysis of Bioactive Compounds from Camellia sinensis L. Extract (CLE) Using UPLC-Q-TOF MS Analysis

The compounds in CLE were analyzed using a UPLC-Q-TOF MS system (Waters, Milford, MA, USA). Compounds were isolated using an Acquity UPLC BEH C_18_ column (Waters, Milford, MA, USA), equilibrated with water containing 0.1% formic acid and eluted with a gradient of acetonitrile containing 0.1% formic acid. Eluted compounds were detected using a Q-TOF MS system with positive/negative electrospray ionization mode using the following instrument settings in the *m*/*z* 50 to 1500 range with scan times of 0.2 s and scan-to-scan delay times of 0.02 s. The capillary voltage was set to 2.5 kV, and the cone voltage was set to 20 V. The nebulized gas was set to 900 L/h at a temperature of 100 °C in positive/negative mode and cone gas flow was set to 30 L/h. All analyses were performed using a locking spray to ensure accuracy and reproducibility. The compounds were identified based on online databases, including the ChemSpider database in UNIFI and the METLIN dataset. Quantitative analysis of the identified compounds was performed using multiple reaction monitoring (MRM) mode of a UPLC-Q-TOF MS system.

### 2.3. Preparation of Cigarette Smoke Extract (CSE)

Research cigarettes 3R4F were purchased from the University of Kentucky (Lexington, KT, USA). For CSE preparation, unfiltered cigarettes were burned, and the smoke was passed through 10 mL of PBS using a vacuum pump for 1 min. We measured the absorbance of CSE filtered at 320 nm using an Epoch microplate reader (BioTek, Winooski, VT, USA) after filtration through a Millex-LCR hydrophilic PTFE 0.45 μm filter (Merck-Millipore, Burlington, MA, USA) and adjusted to an optical density value in the range of 0.9–1.0 for standardization of the CSE. CSE was stored at −80 °C until used in the experiment.

### 2.4. H292 Cell Experiment

#### 2.4.1. Cell Culture

H292 human airway epithelial cells were obtained from ATCC (Manassas, VA, USA). H292 cells were cultured in RPMI 1640 medium containing 10% FBS and 1% penicillin-streptomycin. Cells were incubated at 37 °C in 5% CO_2_ for 24 h.

#### 2.4.2. Cell Viability

A WST-1 (Water-Soluble Tetrazolium 1) assay was performed to evaluate the effects of CLE on cell viability. H292 cells (1 × 10^5^ cells/mL) were incubated in 48 well cell culture plates and were co-treated with 2% CSE and 100 ng/mL lipopolysaccharides (LPS) and CLE (12.5, 25 μg/mL). The following day, supernatants were collected and used to measure the levels of pro-inflammatory cytokines. The WST-1 solution was mixed with cell culture medium at a ratio of 1:10. Next, 300 μL of the mixed medium was added to each well and incubated at 37 °C for 1 h. Absorbance at 450 nm was measured using an Epoch microplate reader (BioTek, Winooski, VT, USA).

#### 2.4.3. ELISA

The cell supernatants were used to measure IL-6, IL-8, and MUC5AC levels using ELISA kits. Briefly, each well was coated overnight with a capture antibody. The following day, each well was washed with a washing buffer and blocked by adding the assay solution for 1 h. Standards and supernatants were diluted with the assay solution and added to the appropriate wells for 2 h. The detection antibody and horseradish peroxidase (HRP) were mixed with reagent diluents were added to the appropriate wells for 1 h. The substrate solution was added to the appropriate wells for 30 min. After 30 min, a stop solution was added to each well. The absorbance was measured at 450 nm using an Epoch microplate reader (BioTek, Winooski, VT, USA).

#### 2.4.4. Quantitative Real-Time RT-PCR

H292 cells (8 × 10^4^ cells/well) were seeded into 12-well plates and incubated at 37 °C for 24 h. The next day, the composition of the cell culture medium was changed the FBS concentration was lowered to 0.2%, and starvation was implemented for 24 h at 37 °C. H292 cells were then co-treated with CLE (12.5, 25 μg/mL), 2% CSE, and 100 ng/mL LPS for 24 h. Total RNA was extracted from H292 cells using the QIAzol^®^ Lysis Reagent. Extracted RNA was quantified using a NanoDrop 2000 spectrophotometer (Thermo Fisher Scientific, Waltham, MA, USA). Extracted RNA was used to synthesize cDNA at 45 °C for 1 h, followed by 95 °C for 5 min, in a C1000^TM^ Thermal Cycler. qRT-PCR was performed using Rotor-Gene SYBR Green Master Mix (QIAGEN, Valencia, CA, USA) on a Rotor-Gene Q real-time PCR system in the presence of gene-specific primers. Information on RNA quality and quantity parameters, and primer sequences are included in the Supplementary Materials.

### 2.5. HL-60 Cell Experiment

#### 2.5.1. Cell Culture

HL-60 cells were obtained from ATCC (Manassas, VA, USA). HL-60 cells were cultured in RPMI 1640 medium containing 10% FBS, and 1% penicillin–streptomycin. Cells were incubated at 37 °C in 5% CO_2_ for 24 h.

#### 2.5.2. Neutrophil Elastase Assay

HL-60 cells (1 × 10^5^ cells/mL) were seeded in 24-well plates and incubated at 37 °C. The cells were then co-treated with CLE (25 and 50 μg/mL) and phorbol myristate acetate (PMA) (100 nM) for 3 h. After 3 h, the HL-60 cells were centrifuged at 3000 rpm for 5 min to obtain supernatants. The supernatants were used to measure neutrophil elastase. Neutrophil elastase level was determined using an ELISA kit (Thermo Fisher Scientific, Waltham, MA, USA), following the manufacturer’s instructions.

#### 2.5.3. NETosis Assay

HL-60 cells (1 × 10^5^ cells/mL) were seeded in 24-well plates and incubated at 37 °C for 30 min. The cells were then co-treated with CLE (25 and 50 μg/mL) and PMA (100 nM) for 3 h. After the SYTOX green dye was diluted (1:500) with Hank’s balanced salt solution (HBSS), it was added to the wells and incubated for 10 min. Fluorescence intensities were measured using a SpectraMax i3 (Molecular Devices, San Jose, CA, USA) at 480/530 nm.

#### 2.5.4. ROS Assay

HL-60 cells (1 × 10^5^ cells/mL) were seeded in a 96-well plate and cultured overnight. The cells were then co-treated with CLE (50 μg/mL) and PMA (100 nM) for 20 min; after 20 min, cells were washed with HBSS. Washed cells were treated with DHR123 5 μM, and incubated for 20 min. Cells were then washed with HBSS, transferred to a 96-well black plate, and absorbance was measured at 500/530 nm using SpectraMax i3 (Molecular Devices, San Jose, CA, USA).

### 2.6. MH-S Cell Experiment

#### 2.6.1. Cell Culture

MH-S mouse alveolar macrophages were purchased from ATCC (Manassas, VA, USA). MH-S were cultured in an RMPI-1640 medium containing 10% FBS, 1% penicillin–streptomycin and 0.05 mM 2-mercaptoethanol. Cells were incubated at 37 °C in 5% CO_2_ and for 24 h.

#### 2.6.2. ELISA

MH-S cells (2 × 10^5^ cells/mL) in 96-well plates were co-treated with 1% CSE, 10 ng/mL LPS, and CLE (25, 50, and 100 μg/mL). The next day, the supernatants were harvested and used to measure IL-6 and macrophage inflammatory protein-2 (MIP-2) levels using an ELISA kit following the manufacturer’s instructions.

#### 2.6.3. NO Assay

To measure the level of NO production, MH-S macrophages (5 × 10^5^ cells/mL) in 48-well plates were co-treated with 1% CSE and 10 ng/mL LPS and CLE (25, 50 and 100 μg/mL) at 37 °C for 24 h. The following day, the supernatants were harvested and used to measure the level of NO production using a nitric oxide assay kit following the manufacturer’s instructions.

#### 2.6.4. Western Blot Assay

MH-S macrophages (5 × 10^5^ cells/mL) were seeded in 6-well culture plates and incubated at 37 °C for 24 h. The next day, MH-S macrophages were treated with CLE (25, 50, and 100 μg/mL) for 2 h prior to the stimulation period with 1% CSE and 10 ng/mL LPS. For protein extraction, MH-S cells were collected in DPBS and lysed in an ice-cold cell lysis buffer containing protease inhibitors. The protein concentration of each sample was measured using the Bradford protein assay. A total 10 μg of the protein mixture from each sample was loaded into wells of Mini-PROTEAN^®^ TGX^TM^ Precast Gels. After electrophoresis, the proteins were transferred onto Western blot filter membranes for 60 min at 100 V. After transfer, the membranes were blocked with EveryBlot blocking buffer (Bio-Rad) for 20 min at room temperature. Then, membranes were incubated with diluted NF-κB p65, IκB, inducible nitric oxide synthase (iNOS), nuclear factor erythroid-2-related factor 2 (NRF2), heme oxygenase-1 (HO-1), lamin B1, and β-actin (Cell Signaling Technology, Danvers, MA, USA) antibodies overnight at 4 °C. The following day, the membranes were washed thrice with TBST for 10 min. After washing, the membranes were incubated with anti-mouse or rabbit IgG antibodies conjugated with HRP for 1 h at room temperature and washed three times with TBST for 10 min. The protein bands were quantified using a ChemiDoc XRS+ (Bio-Rad, Hercules, CA, USA).

### 2.7. Animal Study

Male BALB/C mice (5 weeks old, 20 g) were purchased from Orient Bio (Gyeonggi, Korea). After a 1-week acclimation period, mice were randomly divided into the following four groups: control group (Naïve, *n* = 10), PPE/CSE-treated group (COPD, *n* = 10), PPE/CSE and 200 mg/kg CLE treated group (CLE, *n* = 10), and 10 mg/kg roflumilast-treated group (ROF, *n* = 10), as the positive control. The human effective dose (HED) of CLE 200 mg/kg was calculated as 16.22 mg/kg. Considering the average human body weight is 60 kg, daily administration dosage was 973 mg. The dosage of ROF (10 mg/kg) was decided based on the references [24,25] that indicate ROF effectively attenuated COPD symptoms such as airway inflammation, mucus overproduction, and alveolar destruction. To set the COPD model, mice were intranasally treated with 20 μL of PPE (1.2 U/head) once a week and CSE (100%) three times a week from days 7 to 18. We decided the dose of PPE/CSE by referring to following references presenting typical COPD symptoms such as airway inflammation, mucus overproduction, and emphysema [26,27]. The COPD and CLE groups were given 200 μL/mouse of PBS or CLE diluted in PBS from days 0 to 18. The ROF group was given 200 μL/mouse of roflumilast (ROF) diluted in PBS, orally administered from days 7 to 18. ROF is a drug that acts as a selective, long-acting inhibitor of the enzyme phosphodiesterase-4 (PDE4) for severe COPD associated with chronic bronchitis, and is used after COPD diagnosis due to side effects. Therefore, ROF was treated after inducing COPD. The mice were sacrificed on day 19. BALF, blood, and lung tissue were collected for analysis. All animal procedures were performed in accordance with the animal use and care of the Korea Food Research Institute (approval number: KFRI-M-19001) Animals were maintained in the same room under conventional conditions, with a regular 12 h light/dark cycle, and temperature and relative humidity maintained at 23 ± 2 °C and 50 ± 5%, respectively. Mice were allowed free access to food and water.

### 2.8. Analysis of Cytokines and Chemokines in BALF

Cytokines, such as IL-6, and TNF-α, and chemokines, such as MIP-2, KC, MCP-1 MDC, and TARC in the BALF, were assessed using a Q-plex assay kit (Quansys Biosciences, Logan, UT, USA). Briefly, a calibration curve was generated using a series of 1:2 dilutions with known concentrations of various cytokines and chemokines. Fifty microliters of the calibrator and samples from each well were added to a Q-Plex^TM^ array 96-well plate and incubated for 1 h at room temperature. The plates were then incubated with streptavidin-HRP and the substrate mixture at room temperature for 15 min. Finally, images were generated in the Q-View^TM^ Imager LS using an exposure time of 270 s. Cytokine and chemokine concentrations were analyzed using the Q-View^TM^ software (Quansys Biosciences, Logan, UT, USA).

### 2.9. Analysis of the Immune Cell Counts and Diff-Quick Stain in BALF

To measure the total immune cell count in BALF, 10 μL of BALF were mixed with 10 μL of Accustain T solution (NanoEntek, Seoul, Korea). Then, 12 μL of this solution were loaded into the Accuchip channel and the total cells were counted using the ADAM-MC^TM^ automated cell counter. BALF 150 μL was placed onto the coated slides. Immune cells on the slides were stained using Diff-Quick staining reagent (38721; SYSMEX, Kobe, Japan), and the number of macrophages and neutrophils was counted using a microscope.

### 2.10. Histological Analysis of Lung Tissues

The left-lung samples were immediately fixed in 10% formalin solution by immersion method at room temperature for 3 days. The fixed lung specimens were dehydrated and embedded in paraffin. Lung tissues were sectioned at 4.5 μm thickness. The sections were stained with hematoxylin and eosin (H & E) and periodic acid-Schiff (PAS) and observed under a microscope. We first measured the alveolar mean linear intercept (MLI) as follows: Firstly, select a total of 10 non-overlapping views (1000 μm × 1000 μm) randomly from the suitable areas of 3 lung sections of 3 mice. Place a grid with 10 evenly distributed vertical lines and 10 equally distributed horizontal lines of defined length (1000 µm) on the chosen areas of view using a ruler tool; each line is thus spaced 100 µm apart. The value of one intercept was defined as the linear length between two adjacent alveolar epithelia. MLI is the average value of the intercept lengths which were measured from the total of 10 square views. Additionally, the severity of peri-bronchial inflammation was analyzed semi-quantitatively: 0, no inflammation cells; 1, a few inflammatory cells; 2, a ring of inflammatory cells 1 cell-layer deep; 3, a ring of inflammatory cells 2 cells deep; 4, a ring of inflammatory cells 3–4 cells deep; and 5, a ring of inflammatory cells > 4 cells deep. Finally, we conducted PAS-positive cell count. The lung tissues were stained with PAS stain to evaluate the mucus secretion in which goblet cells play the main role. The goblet cells were stained and appeared with purple color. The number of PAS-positive cells in the bronchus of lung tissues was counted using light microscopy at × 200 magnification, which was based on counting nucleus with dark purple stained cytoplasm. Additionally, area density of PAS positive was measured using FIJI application.

### 2.11. Statistics

All data were analyzed using GraphPad Prism software (version 9.0; La Jolla, CA, USA) and were statistically analyzed using a one-way analysis of variance followed by Dunnett’s post hoc test to compare multiple groups using SPSS software version 20 (IBM SPSS Inc., Armonk, NY, USA). The values for in vitro and in vivo data are expressed as the mean ± standard deviation (SD) of independent experiments. Statistical *p*-value < 0.05 was considered to be statistically significant.

## 3. Results

### 3.1. Effect of CLE on Chronic Obstructive Pulmonary Disease (COPD) Mice

**Figure 1:**
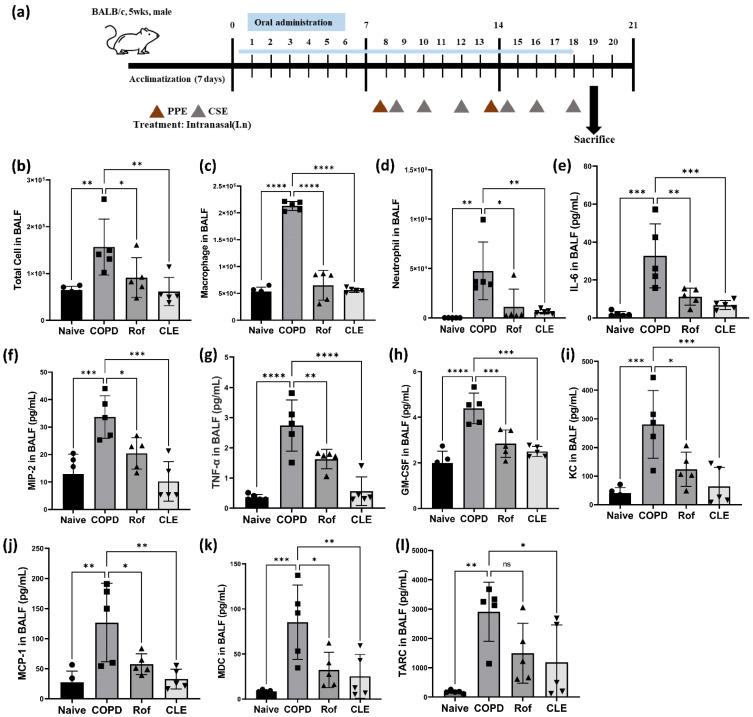
Effect of the CLE on the inflammatory cell count, cytokines and chemokines in BALF from the PPE- and CSE-induced COPD model. (**a**) The timeline of mouse PPE and CSE sensitization and oral administration. (**b**–**d**) Total cell, macrophages and neutrophils numbers in BALF following PPE and CSE treatment. (*n* = 5) (**e**–**l**) BALF levels of IL-6, MIP-2, TNF-α, GM-CSF, KC, MCP-1, MDC and TARC were analyzed by Q-plex assay kit. (*n* = 5) Data were analyzed by one-way ANOVA by Dunnett’s test. All values are reported as mean SD. * *p* < 0.05, ** *p* < 0.01, *** *p* < 0.001, **** *p* < 0.0001. Naïve (Circle): vehicle control, COPD (Square): PPE/CSE treatment, ROF (Triangle): roflumilast (10 mg/kg) + PPE/CSE treatment, CLE (Inverted triangle): *Camelia sinensis* ethanol extract (200 mg/kg) + PPE/CSE treatment. CLE: *Camellia sinensis* L. extract, PPE: Porcine pancreas elastase, CSE: Cigarette smoke extract, COPD: Chronic obstructive pulmonary disease, IL-6: Interleukin 6, MIP-2: Macrophage inflammatory protein-2, TNF-α: Tumor necrosis factor-α, GM-CSF: Granulocyte–macrophage colony-stimulating factor, KC: Keratinocyte-derived chemokine, MCP-1: Monocyte chemoattractant protein-1, MDC: Macrophage-derived chemokine, and TARC: Thymus- and activation-regulated chemokine.

We analyzed inflammatory cells, pro-inflammatory cytokines, and chemokines in BALF, along with histological changes in lung tissue in the PPC/CSE-induced COPD mouse model to determine whether CLE treatment was effective in attenuating lung inflammation. CLE was orally administered on days 0 to 18, and ROF was administrated from day 8 (Figure 1a). The effects of CLE on infiltrating total cells, macrophages, and neutrophils in BALF are shown in Figure 1b–d. Compared to the naïve group, the COPD group showed a marked increased number of total inflammatory cells, macrophages, and neutrophils in BALF, whereas CLE treatment significantly decreased the number of infiltrating cells. Concordantly, levels of pro-inflammatory factors, such as IL-6, MIP-2, TNF-α, GM-CSF, KC, MCP-1, MDC, and TARC, which increased in the COPD group, were significantly decreased in the CLE group (Figure 1e–l).

**Figure 2:**
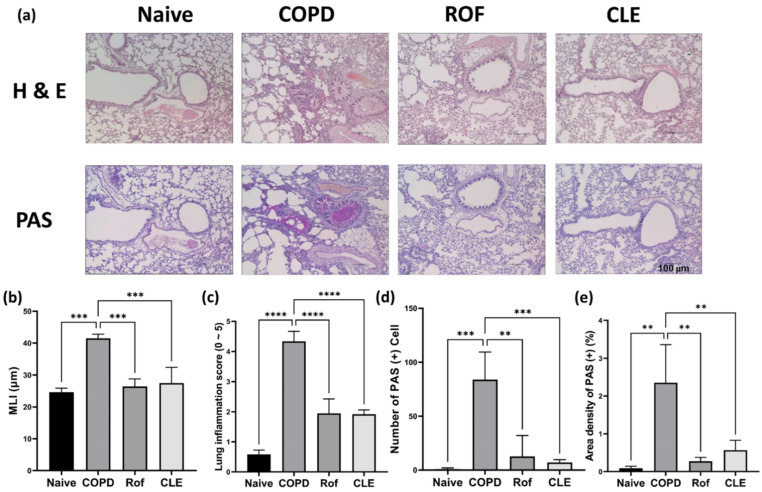
Effect of the CLE on airway inflammation, alveolar enlargement in lung tissues and the mucus production in BALF from the PPE- and CSE-induced COPD. (**a**) Mice were treated with PPE and CSE intranasally in the presence or absence of CLE or ROF treatment. On day 19, lung tissue secretions of each mouse group were stained for hematoxylin and eosin and periodic acid-Schiff to quantify COPD-like histopathological symptoms and mucus production. (*n* = 3) (**b**–**e**) Alveolar airspace enlargement and severity of pulmonary inflammation were assessed using MLI, lung inflammation score, PAS (+) cells count, and area density of PAS (+) (*n* = 3). Data were analyzed by one-way ANOVA by Dunnett’s test. All values are reported as mean SD. ** *p* < 0.01, *** *p* < 0.001, **** *p* < 0.0001. Naïve: vehicle control, COPD: PPE/CSE treatment, ROF: roflumilast (10 mg/kg) + PPE/CSE treatment, CLE: *Camelia sinensis* ethanol extract (200 mg/kg) + PPE/CSE treatment. CLE: *Camellia sinensis* L. extract, PPE: Porcine pancreas elastase, CSE: Cigarette smoke extract, COPD: Chronic obstructive pulmonary disease, MLI: Mean linear intercept, PAS: Periodic acid-Schiff.

Concurrently with BALF analysis, we analyzed the histopathological changes in lung tissue using hematoxylin and eosin (H & E) staining and periodic acid-Schiff (PAS) staining. As shown in Figure 2a, inflammatory cell recruitment, epithelial cell hyperplasia, alveolar destruction, air space enlargement, small airway obstruction, and mucus hypersecretion were induced by PPE/CSE. COPD contributed to the aggravation of airway inflammation and the destruction and enlargement of the alveolar space, which were observed in the COPD group; however, in the CLE and ROF groups, these injuries were attenuated (Figure 2b–c). Additionally, a number of goblet cells and large PAS-positive area were observed in the lung tissues of the COPD group compared with naïve. CLE could effectively inhibit PPE/CSE induced proliferation of airway goblet cells and PAS-positive area (Figure 2d–e). Taken together, these results demonstrate that CLE ameliorates PPE/CSE-induced lung inflammation in mice with COPD.

### 3.2. Effect of CLE on Pro-Inflammatory Cytokines, MUC5AC, and MUC5B in H292

**Figure 3:**
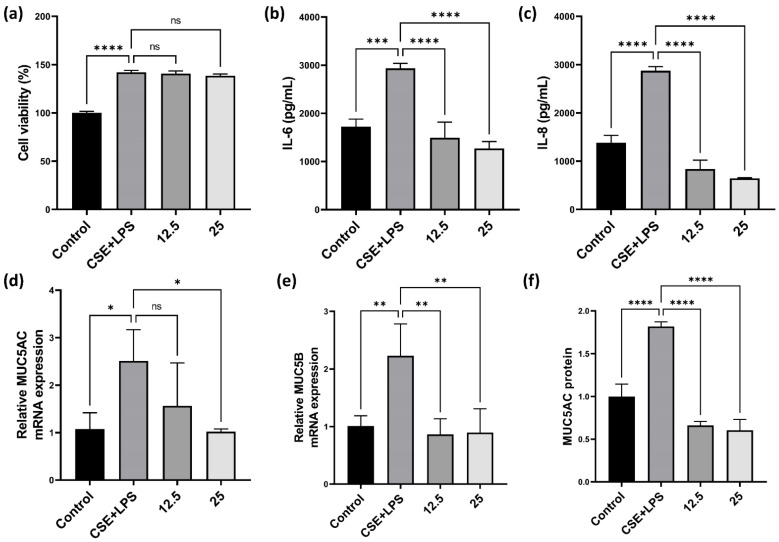
Effect of the CLE on cell viability, the production of cytokines and mucins in H292. (**a**) H292 cells were co-treated with 12.5, 25 μg/mL of CLE and 2% CSE and 20 ng/mL LPS for 24 h. Cell viability was estimated by the WST assay. (**b**,**c**) H292 supernatants were analyzed for IL-6 and IL-8 by ELISA. (**d**,**e**) Total RNA samples were analyzed for MUC5AC and MUC5B mRNA expression by qRT-PCR. (**f**) MUC5AC proteins analyzed by ELISA assay. Data were analyzed by one-way ANOVA by Dunnett’s test. All values are reported as mean SD. * *p* < 0.05, ** *p* < 0.01, *** *p* < 0.001, **** *p* < 0.0001. CLE: *Camellia sinensis* L. extract, CSE: Cigarette smoke extract, LPS: Lipopolysaccharide, IL-6: Interleukin 6, IL-8: Interleukin 8.

As shown by PAS staining (Figure 2a), CLE inhibited airway mucus hypersecretion in PPE/CSE-induced COPD mice. Accordingly, we examined the effects of CLE on the secretion of mucins, the macromolecular components of mucus, and inflammatory cytokines in human airway epithelial cells. H292 cells are often used to elucidate the inflammatory responses involved in airway inflammatory gene expression and mucin secretion. First, we evaluated the cytotoxicity of CLE in H292 cells using the WST assay. As shown in Figure 3a, CLE did not affect the viability of H292 cells at a concentration below 25 μg/mL. Therefore, experiments for IL-6, IL-8, MUC5AC, and MUC5B expression were conducted at CLE concentrations of 12.5, and 25 μg/mL. As indicated in Figure 3d,e, the expression of MUC5AC and MUC5B, the genes encoding the major airway gel-forming mucins, was markedly increased in CSE/LPS-stimulated H292 cells; however, this increase was significantly suppressed in CLE-treated cells. Additionally, as anticipated, MUC5AC protein was significantly increased in CSE/LSP-stimulated H292 cells; this protein increase was significantly suppressed in CLE-treated cells (Figure 3f). In addition, the release of IL-6 and IL-8, the main inflammatory cytokines and chemokines overexpressed in the CSE/LPS group, was reduced in CLE-treated cells in a dose-dependent manner (Figure 3b,c). Our in vitro and PAS staining data showed consistently attenuating effects of CLE on airway epithelial inflammation and mucus hypersecretion.

### 3.3. Effect of CLE on HL-60

As indicated by the BALF data (Figure 1b,e) and H&E staining (Figure 2a), CLE suppressed neutrophil recruitment and immune cell infiltration into the lung tissue. Neutrophils and their products, such as NETS, ROS, and NE, are key mediators of the inflammatory response and airway neutrophilia is a common feature of COPD.

**Figure 4:**
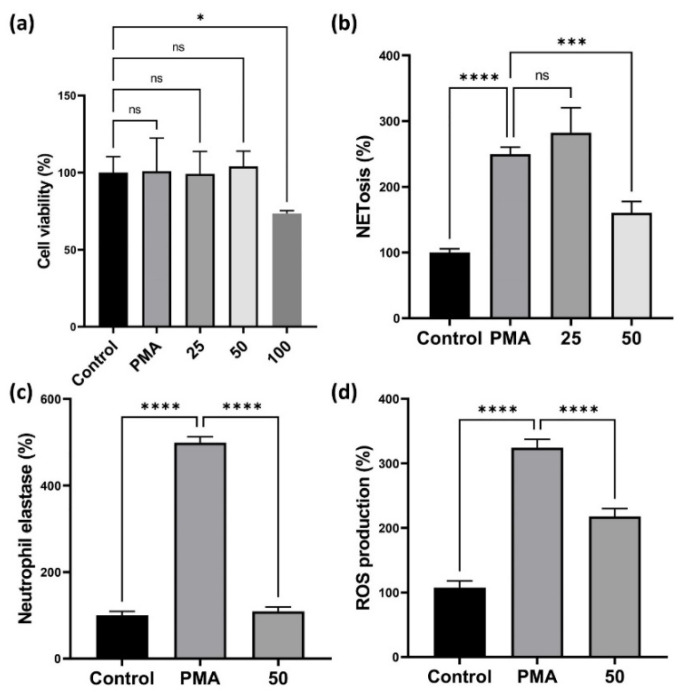
Effect of the CLE on cell viability and the production of NETs, NE, and ROS. (**a**–**c**) The dHL-60 cells were co-treated with 25, 50, and 100 μg/mL of CLE and 100 nM PMA for 3 h. Then, cell viability, NETs, and NE were analyzed. (**d**) For the detection of ROS production, dHL-60 cells were co-treated with 50 μg/mL of CLE and 100 nM PMA for 20 min. Data were analyzed by one-way ANOVA by Dunnett’s test. All values are reported as mean SD. * *p* < 0.05, *** *p* < 0.001, **** *p* < 0.0001. CLE: *Camellia sinensis* L. extract, NETs: Neutrophil extracellular traps, NE: Neutrophil elastase, PMA: Phorbol 12-myristate 13-acetate, ROS: Reactive oxygen species.

Accordingly, we investigated the effects of CLE on neutrophil activation by measuring NETs, ROS, and NE production in PMA-stimulated HL-60 cells. First, we evaluated the cytotoxicity of CLE in HL-60 cells using the WST assay. As shown in Figure 4a, no cytotoxicity was observed up to a CLE concentration of 50 μg/mL. NETs and NE production was measured in subsequent experiments. CLE at 50 μg/mL reduced the production of NETs and NE (Figure 4b,c). As NET release occurs downstream of ROS generation, the ability of HL-60 cells to produce ROS after PMA stimulation was tested in subsequent experiments. In the NET- and NE-producing ability test, only 50 μg/mL CLE reduced NET production, so only 50 μg/mL CLE was used. PMA-stimulated HL-60 cells showed increased ROS production, whereas the production of ROS decreased in HL-60 treated with 50 μg/mL CLE (Figure 4d).

### 3.4. Effect of CLE on Pro-Inflammatory Cytokines and Chemokines in MH-S

**Figure 5:**
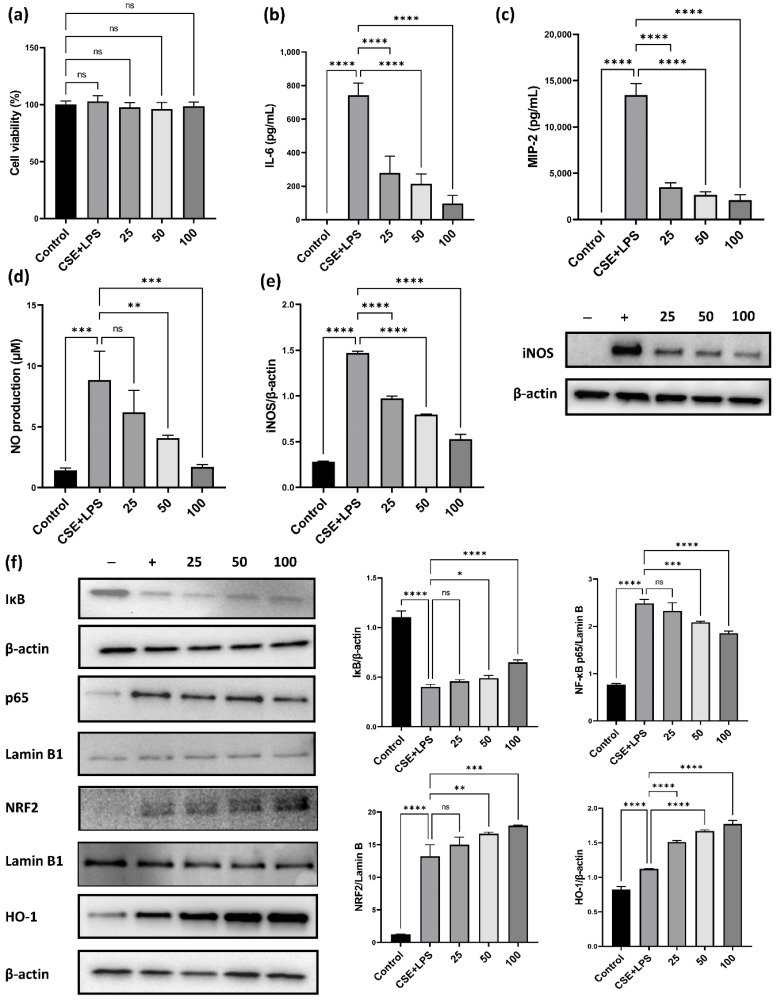
Effect of the CLE on cell viability and the production of cytokines, NO and the activation of NF-κB and NRF2 pathways in MH-S macrophages. For the cell viability assay, (**a**) the MH-S macrophages were co-treated with 25, 50, 100 μg/mL of CLE and 1% CSE and 10 ng/mL LPS for 24 h. Cell viability was estimated by the WST assay. Then, IL-6 (**b**) and MIP-2 (**c**) in the culture supernatants were quantified using ELISA assays. NO production levels (**d**) were determined using Griess assay. (**e**,**f**) Protein expression levels of iNOS, IκB, NF-κB p65, NRF2, HO-1, β-actin, and Lamin B1 were visualized using Western blots. Data were analyzed by one-way ANOVA using Dunnett’s test. All values are reported as mean SD. * *p* < 0.05, ** *p* < 0.01, *** *p* < 0.001, **** *p* < 0.0001. CLE: *Camellia sinensis* L. extract, NO: Nitric oxide, NF-κB: Nuclear factor-κB, NRF2: Nuclear factor-E2-related factor 2, CSE: Cigarette smoke extract, LPS: Lipopolysaccharide, IL-6: Interleukin 6, MIP-2: Macrophage inflammatory protein-2, iNOS: Inducible nitric oxide synthase, HO-1: Heme oxygenase-1.

As shown in Figure 1b,e, CLE markedly inhibited the recruitment of macrophages which play a critical role in chronic pulmonary inflammation induced by PPE/CSE. Accordingly, we investigated the anti-inflammatory and antioxidant effects of CLE on CSE/LPS-stimulated MH-S alveolar macrophages. First, we evaluated the cytotoxicity of CLE on MH-S cells using the WST assay. As shown in Figure 5a, CLE did not affect the viability of MH-S cells at a concentration below 100 μg/mL, while a cytotoxic effect was observed at 200 μg/mL. Therefore, experiments for IL-6, MIP-2, NO, iNOS, NF-κB, NRF2, and HO-1 were conducted at 25, 50, and 100 μg/mL CLE. As described in Figure 5c–e, IL-6, MIP-2, and NO secretion levels were markedly increased in CSE/LPS-stimulated macrophages; however, in CLE-treated MH-S, this hypersecretion was inhibited in a dose-dependent manner. Similarly, iNOS protein overexpression in CSE/LPS was reduced by CLE in a dose-dependent manner (Figure 5e). Consequently, we investigated the effect of CLE on NF-κB, a transcription factor involved in the expression of pro-inflammatory mediators such as IL-6, MIP-2, and NO. As shown in Figure 5f, CSE/LPS stimulation induced IκB degradation in the cytoplasm and NF-κB p65 accumulation in the nucleus of MH-S cells, indicating that NF-κB was activated. CLE suppressed CSE/LPS-induced degradation of cytosolic IκB and NF-κB p65 nuclear translocation in a dose-dependent manner. Taken together, these findings suggest that the anti-inflammatory effect of CLE is mediated by the suppression of NF-κB p65 translocation and IκB degradation, resulting in the inhibition of the NF-κB signaling pathway in CSE/LPS-stimulated macrophages.

To confirm whether the anti-inflammatory effect of CLE on CSE/LPS-induced inflammation was related to the NRF2/HO-1 pathway, a major antioxidant defense system that suppresses the excessive ROS production and NF-κB activation, we analyzed the protein expression of NRF2 and HO-1. As anticipated, NRF2 and HO-1 levels were markedly increased in CSE/LPS-stimulated MH-S cells; however, this increase was much higher in MH-S cells co-treated with CSE/LPS and CLE, and the increase was dose-dependent. These findings suggest that the anti-inflammatory effect of CLE is associated with the activation of NRF2/HO-1.

### 3.5. Phytochemical Constituents of CLE

**Figure 6:**
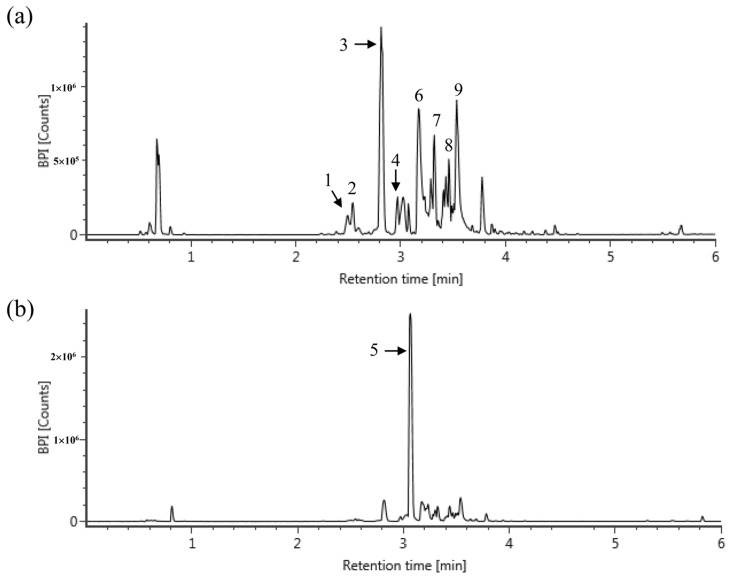
Representative mass chromatograms of CLE. The mass chromatograms were obtained using UPLC-Q-TOF-MS with positive (**a**) and negative (**b**) mode, and nine compounds were identified. (1) Gallocatechin (GC); (2) Epigallocatechin (EGC); (3) GC dimer; (4) catechin; (5) caffeine; (6) Epigallocatechin gallate (EGCG); (7) Quercetin 3-glucosylrutinoside; (8) kaempferol 3-glucosylrutinoside; (9) Catechin gallate (GC).

The chemical profiles of CLE were analyzed using UPLC-Q-TOF-MS with positive and negative mode, and nine major compounds were identified as gallocatechin (GC), epigallocatechin (EGC), GC dimer, catechin, caffeine, epigallocatechin gallate (EGCG), quercetin 3-glucosylrutinoside, kaempferol 3-glucosylrutinoside, and catechin gallate (CG) (Figure 6). Their contents in CLE were measured using the MRM mode of UPLC-Q-TOF-MS (Table 1). The MS data revealed that caffeine analyzed using the negative mode was the most abundant compound in CLE, and its amount in CLE was 70.09 mg/g of CLE. Caffeine was followed by GC dimer, EGCG, CG, quercetin 3-glucosylrutinoside, kaempferol 3-glucosylrutinoside, GC, catechin, and EGC with the contents of 41.88, 38.72, 20, 6.96, 5.91, 3.42, 2.67, and 1.14 mg/g of CLE, respectively.

## 4. Discussion

COPD is one of the most common pulmonary diseases and is characterized by progressive airflow limitation and recurrent inflammation [28,29]. The repeated inflammatory response disrupts normal repair and defense mechanisms, leading to mucus hypersecretion (chronic bronchitis), tissue destruction (emphysema), fibrosis, and small-airway inflammation (bronchitis) [6]. The pathogenesis of COPD mainly involves inflammation, oxidative stress, and protease balance. In particular, several cell types such as airway epithelial cells, neutrophils, and alveolar macrophages are implicated in the pathogenesis of COPD.

In the airway epithelium, repeated inflammation causes extensive metaplasia of bronchial epithelial goblet cells and hypertrophy and proliferation of submucosal bronchial cells, which then accelerate obstructions of the airway and reduce pulmonary function [6,7]. The regulation of mucus secretion could be an important target for ameliorating COPD. As shown in Figure 2a,d,e, PPE and CSE treatment promoted mucus production. We confirmed that the expression of mucins *MUC5AC*, *MUC5B* gene and MUC5AC protein was increased by CSE/LPS stimulation in airway epithelial H292 cells (Figure 3d–f). Mucus hypersecretion has been reported to increase airway resistance. Mice exposed to cigarette smoke for 8 weeks increased hysteresis, transpulmonary pressure, and airway-specific resistance [30]. In our study, we confirmed that administration of CLE decreased mucus hypersecretion in a mouse model of COPD, and decreased the mRNA levels of *MUC5AC*, *MUC5B* and MUC5AC protein production in in vitro experiments. Taken together, these results suggest that CLE intake may alleviate airway resistance caused by mucus hypersecretion in COPD patients.

Neutrophilic inflammation is a prominent feature of COPD, and the neutrophils are key effector cells in this disease. In patients with COPD, increased numbers of activated neutrophils have been found in sputum and BALF [31,32]. The most common feature of neutrophilic inflammation in COPD is the formation of NETs. In general, NET formation represents a specialized host defense mechanism that entraps and eliminates invading microbes. NETs are web-like scaffolds of extracellular DNA in complex with histones and anti-microbial neutrophil granular proteins, such as matrix metalloproteinase (MMP) and NE [33]. However, NETosis in COPD contributes to alveolar destruction through the secretion of NE, cathepsin G, proteinase 3, MMP-8, and MMP-9 leading to a decline in lung function [34,35]. In the present study, HL-60 cells were used to analyze the NET-forming NETosis reactions. As shown in Figure 4b, PMA stimulation increased NETosis in HL-60 cells. Since NET formation by NETosis depends on intracellular ROS, we analyzed the production of intracellular ROS, and then CLE treatment decreased their production (Figure 4c). Therefore, we suggest that CLE treatment may inhibit NETosis products such as NE by reducing ROS generation, resulting in the reduction in neutrophil infiltration (Figure 1e).

Numerous studies have highlighted that alveolar macrophages play a pivotal role in COPD from a pathophysiological perspective. In patients with COPD, the number of alveolar macrophages increases in various samples, such as airways, lung parenchyma, BALF, and sputum [36,37,38]. This increase was also observed in our results (Figure 1d). In COPD, activated alveolar macrophages release inflammatory mediators including IL-6, TNF-α, CXCL1 (KC), MIP-2, CXCL6, CXCL8, CCL2, and leukotriene B4 (LTB4). These mediators are particularly efficient in recruiting other innate immune cells, such as neutrophils, that remove pathogens [39]. In particular, KC and MIP-2 can recruit neutrophils into the lung (inflammatory regions) (Figure 1e). Our data showed increased KC and MIP-2 in COPD mice (Figure 2c–f) are suppressed by CLE treatment (Figure 2c–f). It is thought that these suppressed chemokines participated in inhibiting the infiltration of neutrophils into the BALF (Figure 1e).

ROF is the only oral drug approved by the European Union in June 2010 for the treatment of COPD. Since our experimental design was to evaluate the efficacy of oral administration of CLE, we used ROF as a positive control. Cyclic adenosine monophosphate (cAMP) is a second messenger that affects homeostasis and cellular functions, such as structure and inflammatory cells, and is unstable and rapidly hydrolyzed by PDEs [40]. Therefore, the inhibition of PDE leads to physiological changes such as increased intracellular cAMP and increased smooth muscle relaxation and inhibition of inflammation. ROF is known to inhibit the release of inflammatory cytokines and inflammatory mediators, inhibit neutrophil migration and activity, and promote apoptosis of inflammatory cells through PDE-4 inhibition. In our study, ROF administration also inhibited the infiltration of inflammatory cells such as neutrophils and macrophages in the BALF, and suppressed the secretion of inflammatory cytokines and chemokines, but was less effective than CLE. Although, the administration period of ROF was 1 week shorter than that of CLE due to the side effects of ROF such as weight loss, diarrhea, nausea, and insomnia, but considering that ROF is a therapeutic agent, the activity of CLE seems to be better than that of the ROF. Moreover, referring to safety studies on green tea and its main bioactive components, CLE (200 mg/kg) used in this study is relatively free from the concerning side-effects issue compared to ROF, so its use in prophylactic or therapeutic aspects is expected. These attenuating effects of CLE on CSE/PPE-induced lung inflammation can be elucidated by mechanisms that CLE inhibits the NF-κB p65 translocation and activation of NRF2/HO-1.

NF-κB, a family of inducible transcription factors, regulates a large array of genes involved in immune and inflammatory responses [41]. In alveolar macrophages, NF-κB activation can induce the production of IL-6, TNF-α, IL-1β, MIP-2, and iNOS. In the present study, CSE and LPS treatment increased the production of IL-6, TNF-α, MIP-2, iNOS, and NO (Figure 5b–e). Furthermore, treatment with CSE and LPS activated the NF-κB pathway by degrading IκB in the cytosol and translocating p65 into the nucleus. In contrast, CLE induced the inactivation of NF-κB (Figure 5f), resulting in the suppression of the production of pro-inflammatory cytokines, chemokines, iNOS, and NO (Figure 5b–e). Another crucial pathway is NRF2/HO-1, an antioxidant defense system. NRF2 is located in the cytoplasm where it is bound to the inhibitory protein Keap1 under normal physiological conditions [42]. Under oxidative stress, NRF2 is released from Keap1, and translocated into the nucleus. Next, NRF2 binds to antioxidant response elements (ARE) to regulate the transcription of antioxidant genes such as the inducible enzyme HO-1 [43]. HO-1 plays a pivotal role in host defense against oxidative stress [44]. Therefore, the NRF2/HO-1 pathway was investigated to confirm the anti-inflammatory and antioxidant effects of CLE. We analyzed the expression of NRF2 and HO-1 after CLE treatment in CSE- and LPS-stimulated MH-S. As shown in Figure 5f, the CLE induced NRF2 translocation in a dose-dependent manner. Accordingly, HO-1 production increased in a dose-dependent manner following CLE treatment. Based on the above results, we suggest that CLE treatment induced the activation of the NRF2/HO-1 signaling pathway and that the NF-κB/IL-6/MIP-2 signaling pathway was suppressed by CLE.

Tea, extracted from *Camellia sinensis* L., is one of the most widely consumed drinks in the world [45]. Tea is an evergreen plant that mainly grows in the tropical and temperate regions of Asia, including China, India, Sri Lanka, Japan, and Korea [45]. Tea from the plant *Camellia sinensis* is consumed as green, black, or oolong tea in many parts of the world. It is estimated that approximately 2.5 million tons of tea leaves are produced worldwide annually, of which 20% are produced as green tea, mainly consumed in Asia, parts of North Africa, the United States, and Europe [14]. The association between tea consumption, particularly green tea, and human health has long been recognized [46,47]. Green tea contains polyphenols, including flavanols, flavanediols, flavonoids, and phenolic acids. These components may constitute up to 30% of a tea leaf [47]. Most green tea polyphenols are flavonols, commonly known as catechins, including epicatechin, epigallocatechin, epicatechin-3-gallate, and epigallocatechin gallate (EGCG) [48]. A typical 250 mL of green tea beverage contains 50–100 mg of catechins [49]. Considering studies that daily consumption of tea containing 690 mg catechins for 12 weeks reduced body fat and showed antioxidant effects [50], the proper intake to reap health benefits is calculated as 1750–3500 mL of green tea per day. For this reason, there is a need for an extraction method that can more effectively ingest the useful components of green tea. According to a recent study analyzing the type and content of catechins between water extraction and ethanol extraction, there was no difference in the types of major catechins detected, but the catechins content increased even more when the 25–95% ethanol extraction was performed more than the water extraction [51]. Therefore, we extracted the CLE using 70% ethanol. Concordant with previous reports on compositions of CN, catechins such as gallocatechin dimer, epigallocatechin gallate (EGCG), and catechin gallate were detected in the CLE used in this study. The well-known functions of these catechins are antioxidant and anti-inflammatory activities. The gallocatechin dimer showed anti-inflammatory and antioxidant effects by reducing the synthesis of prostaglandin E2 (PGE2) and also inhibited purified cyclooxygenase-1 (COX-1) and cycloocygeanse-2 (COX-2) [52]. EGCG pre-treatment alleviates LPS-induced acute lung injury (ALI) in mice through the inhibitory effect of protein kinase C alpha (PRKCA) on pro-inflammatory cytokine release through mitogen-activated protein kinase (MAPK) signaling pathways [53]. Additionally, EGCG showed radioprotective efficacy in ^60^Coγ radiation-induced injury mice through immunomodulatory and antioxidant activity [54]. Catechin gallate directly interacts with DNA oligomers and inhibits the activity of COX-1 and COX-2 enzymes, the gene expression of matrix metalloproteinase-9 in macrophage-differentiated HL-60 cells [55]. Interestingly, the CLE contained the caffeine, quercetin 3-glucosylrutinoside, and kaempferol 3-glucosylrutinoside. It was reported that caffein has a protective effect against acute lung injury, and its mechanism was mediated by A_2A_-receptor-independent signals [56]. Based on these findings and references, it is assumed that the anti-inflammatory and anti-oxidative effects of CLE are attributed to catechins, caffeine, quercetin 3-glucosylrutinoside, and kaemperol 3-glucosylrutinoside.

Although our observations presented the attenuating effect of CLE on PPE/CSE-induced lung inflammation, our study has some limitations. The first limitation of our experiment was that we did not confirm an anti-inflammatory and antioxidant mechanism using lung tissue. Next, the forced expired volume (FEV)/the forced vital capacity (FVC) ratio, one of the diagnostic criteria of COPD, was not measured using a respiratory-function-measuring instrument such as flexivent (SCIREQ Inc., Montreal, QC, Canada) in this study. We think it would have been a better study if it was confirmed that the treatment of CLE increased the FEV value. Therefore, in the further study, we will confirm the mechanism in lung tissue of CLE and investigate the attenuating effects and mechanisms of active components from CLE on the responses of inflammation and oxidative stress in pulmonary inflammation models. There is a large body of research on the anti-inflammatory and antioxidant effects of bioactive components from CN; however, studies on attenuating effect for respiratory diseases, especially COPD, have not been reported using these bioactive components. Therefore, if we confirm that oral administration of the bioactive component from CLE improves the lung function, it will be a meaningful follow-up study.

## 5. Conclusions

Oral administration of CLE attenuated COPD symptoms, including the infiltration of inflammatory cells, the increase in inflammatory cytokines and chemokines, mucus hypersecretion, and damage of lung parenchyma in PPE/CSE-induced COPD mice. Concordant with in vivo results, the attenuating effect of CLE on pulmonary inflammation was also confirmed in airway epithelial cells, alveolar macrophages, and neutrophils. In particular, the CLE suppressed the production of inflammatory mediators by inhibiting NF-κB p65 nuclear translocation via activation of the NRF2/HO-1 pathway in CSE/LPS-stimulated alveolar macrophages. Therefore, CLE administration may be an effective approach for treating or preventing chronic pulmonary diseases such as COPD induced by cigarette smoke.

## Figures and Tables

**Table 1:** Identification of major compounds from CLE using UPLC-Q-TOF MS with MRM mode and their contents.

	RT ^(a)^	Compounds	Exact Mass (*m*/*z*) [M − H] or [M + H]	MS Fragments	Content (mg/g of CLE)
1	2.5	epigallocatechin	305.0660	125, 137, 219	1.14
2	2.54	gallocatechin	305.0660	125, 137, 219	3.42
3	2.81	gallocatechin dimer	611.1426	219, 305	41.88 ^(b)^
4	2.97	catechin	289.0708	245	2.67
5	3.07	caffeine	195.0899	138	70.09
6	3.18	epigallocatechin gallate	457.0777	169, 305	38.72
7	3.32	quercetin 3-glucosylrutinoside	771.1975	316, 413, 593	6.96 ^(b)^
8	3.46	kaempferol 3-glucosylrutinoside	755.2038	285, 463	5.91 ^(b)^
9	3.54	catechin gallate	441.0830	169, 289	20.00

^(a)^ RT, retention time; ^(b)^ the contents of gallocatechin dimer, quercetin 3-glucosylrutinoside, and kaempferol 3-glucosylrutinoside were expressed as gallocatechin, quercetin, and kaempferol equivalents, respectively.

## Data Availability

The data are contained within the article.

## Supplementary Materials

The following supporting information can be downloaded at: https://www.mdpi.com/article/10.3390/antiox11091683/s1, Table S1: Evaluation of quality and quantity parameters of RNA samples. Table S2: A set of primers of Mucin.
