# Supplementary material for: Camellia sinensis L. Alleviates Pulmonary Inflammation Induced by Porcine Pancreas Elastase and Cigarette Smoke Extract
Source: Antioxidants (Basel). 2022 Aug 28;11(9):1683. doi: 10.3390/antiox11091683 (PMC9495585; doi:10.3390/antiox11091683)
Supplement: Supplementary file 1 [file antioxidants-11-01683-s001.zip › antioxidants-1818567-supplementary.pdf]

Table S1. Evaluation of quality and quantity parameters of RNA samples

Sample	260/2680	RNA (ng/μL)	600 ng (μL)	DW (μL)
**CON1**	**2.075**	**110.132**	**5.4**	**14.6**
**CON2**	**2.111**	**120.855**	**5.0**	**15.0**
**CON3**	**2.1**	**117.165**	**5.1**	**14.9**
**CON4**	**2.084**	**92.753**	**6.5**	**13.5**
**CSE+LPS1**	**2.114**	**126.913**	**4.7**	**15.3**
**CSE+LPS2**	**2.072**	**93.456**	**6.4**	**13.6**
**CSE+LPS3**	**2.092**	**103.845**	**5.8**	**14.2**
**CSE+LPS4**	**2.081**	**96.319**	**6.2**	**13.8**
**CLE 12.5-1**	**2.085**	**84.154**	**7.1**	**12.9**
**CLE 12.5-2**	**2.073**	**113.517**	**5.3**	**14.7**
**CLE 12.5-3**	**2.096**	**98.36**	**6.1**	**13.9**
**CLE 12.5-4**	**2.067**	**79.728**	**7.5**	**12.5**
**CLE 25-1**	**2.07**	**89.1**	**6.7**	**13.3**
**CLE 25-2**	**2.093**	**91.406**	**6.6**	**13.4**
**CLE 25-3**	**2.087**	**82.098**	**7.3**	**12.7**
**CLE 25-4**	**2.119**	**86.301**	**7.0**	**13.0**

Table S2. A set of primers of Mucin

	Primer sequence	Annealing Temperature (℃)	Cycle	Amplicon size
MUC5AC (Accession : KC800812)	Forward, 5′-CCACTGGTTCTATGGCAACACC-3′ Reverse, 5′-GCCGAAGTCCAGGCTGTGCG-3′	60 ℃	40	313
MUC5B (Accession : NM_002458.3)	Forward, 5′-CTGCTACGACAAGGACGGAAAC-3′ Reverse, 5′-AAGGCTGTGAGCGCACTGGATG-3′	60 ℃	40	112
SDHA (Accession : KR710499.1)	Forward, 5′-TGGGAACAAGAGGGCATCTG-3′ Reverse, 5′-CCACCACTCATCAAATTCATG-3′	60 ℃	40	85
